# An LC-MS/MS Method to Measure *S*-Methyl-l-Cysteine and *S*-Methyl-l-Cysteine Sulfoxide in Human Specimens Using Isotope Labelled Internal Standards

**DOI:** 10.3390/molecules24132427

**Published:** 2019-07-02

**Authors:** Tharsini Sivapalan, Antonietta Melchini, Jack Coode-Bate, Paul W. Needs, Richard F. Mithen, Shikha Saha

**Affiliations:** 1 Quadram Institute Bioscience, Norwich NR4 7UQ, UK; 2 Department of Urology, Norfolk and Norwich University Hospitals NHS Foundation Trust, Norwich NR4 7UY, UK

**Keywords:** *S*-methyl-l-cysteine, *S*-methyl-l-cysteine sulfoxide, sulphur compounds, liquid chromatography tandem mass spectrometry, body fluids

## Abstract

This is the first report describing an analytical method for quantitative analysis of two naturally occurring sulphur compounds, *S*-methyl-l-cysteine (SMC) and *S*-methyl-l-cysteine sulfoxide (SMCSO), in human body fluids using isotope-labelled internal standards and liquid chromatography-mass spectrometry (LC-MS)/MS techniques. This method was validated according to the guideline of the Royal Society of Chemistry Analytical Methods Committee. It offers significant advantages including simple and fast preparation of human biological samples. The limits of detection of SMC were 0.08 µM for urine and 0.04 µM for plasma. The limits of detection of SMCSO were 0.03 µM for urine and 0.02 µM for plasma. The calibration curves of all matrices showed linearity with correlation coefficients r^2^ > 0.9987. The intra and inter day precisions in three levels of known concentrations were >10% and >20%, respectively. The quantification accuracy was 98.28 ± 5.66%. The proposed method would be beneficial for the rapid and accurate determination of the SMC and SMCSO in human plasma and urine samples using by isotope labelled internal standards.

## 1. Introduction

Dietary sulphur compounds are of potential value in protecting humans against chronic diseases [1,2,3]. The literature is particularly rich in studies supporting the anticancer properties of cruciferous vegetables. These are uniquely characterised by high levels of sulphur-delivering compounds such as isothiocyanate precursors [4,5]. There is growing interest in the bioactivity of *S*-methyl-l-cysteine sulfoxide (SMCSO) [6,7,8,9], which is mainly found in *Allium* species (family: Liliaceae) [10,11,12]. SMCSO is also present in cruciferous vegetables [6,13] and its use as a urinary biomarker of the consumption of these vegetables has been recently suggested [14]. To understand the association between SMCSO intake from diets and its potential benefits for human health, evidence-based knowledge on its bioavailability, including tissue distribution, is required. Several analytical methods have been reported for the analysis of SMCSO in plant materials [15,16,17,18,19,20,21,22], but there are only a few reports on samples of human origin [14,23]. SMCSO content in plant material can be determined by using nuclear magnetic resonance (NMR) spectroscopy [14], gas chromatography-mass spectrometry (GC-MS) [20,21,22,24], high-performance liquid chromatography (HPLC) [17,25], liquid chromatography-mass spectrometry (LC-MS) [26,27,28,29], capillary electrophoresis [16,19], and direct analysis in real time mass spectrometry (DART-MS) [15,30]. These published methods based on GC-MS, HPLC-MS and LC-MS require a derivatisation step prior to MS detection with sample run time between 40–70 min [31]. This represents a major limitation for studies with large sample size. Capillary electrophoresis may represent an alternative method with short run time (~20 min) but it does lack accuracy [31]. DART-MS has also been used to identify SMCSO in plants with good reproducibility but is not suitable for quantitative analysis [15,30]. To the best of our knowledge, only two studies describe SMCSO detection in biological matrices of human origin [14,23]. Karim and colleagues have quantified urinary SMCSO levels following administration of an oral dose of *S*-carboxymethyl-l-cysteine (SCMC) using HPLC with electrochemical detection (ECD) [23]. More recently, a study carried out by Edmands has identified SMCSO, and its structurally related metabolites, in urine samples collected during a three-phase dietary intervention study in twenty subjects [14]; however, SMCSO analyses were performed using NMR which doesn’t provide quantitative data.

Furthermore, only a few studies have described the measurement of its reduced form (*S*-methyl-l-cysteine, SMC). SMC analysis has been reported using LC-MS [32,33], GC-MC [34] and HPLC/ECD [23].

We aimed to establish an analytical method that could allow qualitative and quantitative analyses of SMC and SMCSO with (i) easy sample preparation, (ii) high sensitivity/specificity, (iii) potential application to different biological matrices and (iv) suitability for large sample cohorts. Here we report the development and validation of a rapid, highly sensitive LC-MS/MS method without derivation steps to enable the identification and quantification of SMC and SMCSO in body fluids. This method could represent a valuable analytical tool for carrying out targeted analyses in samples obtained from large dietary intervention studies for better understanding the pharmacokinetics of these compounds in humans.

## 2. Results

### 2.1. Synthesis of Internal Standards

**Figure 1:**
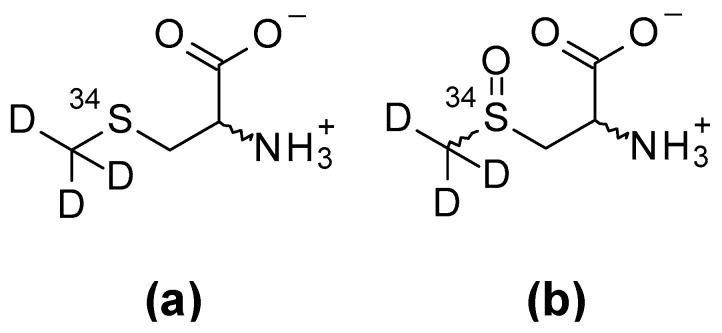
Structures of internal standards (**a**) ^34^*S*-Trideuteromethylcysteine (^34^*S*-d_3_SMC) and (**b**) ^34^*S*-trideuteromethylcysteine sulfoxide (^34^*S*-d_3_SMCSO).

Racemic ^34^*S*-Trideuteromethylcysteine (^34^*S*-d_3_SMC) and racemic, diastereomeric ^34^*S*-trideuteromethylcysteine sulfoxide (^34^*S*-d_3_SMCSO) were synthesised in house; the structure of these compounds is shown in Figure 1.

#### 2.1.1. Identification of ^34^*S*-Trideuteromethylcysteine by 1H-NMR/δ (D_2_O) and Mass Spectroscopy

3.94, dd, *J* = 4.2, 7.8 Hz, 1H, H-α; 3.10, dd, *J* = 4.2, 14.9 Hz, 1H, H-β; 3.00, dd, *J* = 7.8, 14.9 Hz, 1H, H-β’. ESI-MS-*m/z* + 141 [M + H]^+^, 124 [M-17]^+^, loss of OH^−^, 163 [M + Na]^+^.

#### 2.1.2. Identification of ^34^*S*-Trideuteromethylcysteine Sulfoxide by 1H-NMR/δ (D_2_O) and Mass Spectroscopy

First diastereomer −4.29, dd, *J* = 3.84, 8.07, 1H, H-α; 3.42, dd, *J* = 8.07, 14.58, 1H, H-β; 3.42, dd, *J* = 3.84, 14.58, 1H, H-β’. Second diastereomer- 4.23, dd, *J* = 5.97, 7.77, 1H, H-α; 3.50, dd, *J* = 5.97, 13.98, 1H, H-β; 3.25, dd, *J* = 7.77, 13.98, 1H, H-β’. ESI-MS-*m/z* +157 [M + H]^+^, 179 [M + Na]^+^, 88 [M-(D_3_C^34^SO^−^)]^+^.

### 2.2. Optimization of Mass Spectroscopy Conditions

The automated Agilent MassHunter Optimizer software was used to obtain precursor and products ions of SMC, SMCSO, ^34^*S*-d_3_SMC and ^34^*S*-d_3_SMCSO in the electrospray mode. The collision energy was used from 0 to 80 by 10 CE step increment in negative and positive polarity modes. The fragmentor value was constant at 380 V. The positive polarity has produced two more intense product ions (119 and 47) for SMC, three more intense products ions (88, 70 and 42) for SMCSO, two product ions (124 and 42) for ^34^*S*-d_3_SMC and two product ions (88 and 42) for ^34^*S*-d_3_SMCSO. Two of the product ions of SMCSO (88 and 70) have been mentioned in the previous published article [26]. The precursor ion and the product ion with the highest signal-to noise (S/N) value and the highest peak intensity was selected for the quantifier ion and the other two product ions were selected for the qualifier ions. Table 1 summarizes the monitored ions and the optimized MS operating parameters of the analytes and internal standards.

### 2.3. Optimization of LC Parameters

Waters Acquity UPLC HSS C18, Kinetex-C18 1.7 µm (100 mm × 2.1 mm) and Zorbax SB-AQ-C18 1.7 µm (100 mm × 2.1 mm) were used to achieve an optimal retention of SMCSO in these columns by using different mobile phases at different pH values. We found that the use of a Zorbax SB-AQ- C18 1.7 µm (100 mm × 2.1 mm) column and guard column from Agilent^®^ allow good retention, but the peak shape was not satisfactory using mobile phase 0.1% formic acid in water and acetonitrile; good retention and peak shape was obtained by using 10 mM ammonium acetate and 0.05% HFBA in water and in 90% methanol. The methods previously reported by Bernaert [26] and Kim [27] are based on the use of C18 column with 0.1% formic acid as an additive for SMC and SMCSO analysis in plant extract. We achieved better peak shape and sensitivity by applying HFBA on Zorbax SB-AQ-C18 1.7 µm (100 mm × 2.1 mm) column in biological matrices analysis.

### 2.4. Method Validation

Published acceptance criteria for linearity, accuracy, precision, recovery and sample stability were followed for validation of this method [35]. We performed method validation procedures in plasma and urine samples by choosing an appropriate matrix [36].

#### 2.4.1. Linearity and Sensitivity

Calibration curves were linear over a wide range of concentrations for SMC (urine: 0–739.75 µM; plasma: 0–73.98 µM) and for SMCSO (urine: 0-661.46 µM; plasma: 0–66.15 µM). The least-squares regression calibration curve was r^2^ = 0.9995 for urine and r^2^ = 0.9987 for plasma (Table 2). Limit of detection (LOD) and Limit of Quantitation (LOQ) values for SMC and SMCSO are shown in the validation data (Table 2).

#### 2.4.2. Precision and Accuracy

Intraday precision was evaluated by replicate (n = 5) analysis of three levels (low, medium and high) of known concentration spiked in urine (SMC, 1.18 µM, 29.59 µM and 739.75 µM; SMCSO, 1.06 µM, 26.46 µM, 661.46 µM) and plasma (SMC, 1.18 µM, 5.92 µM and 29.59 µM; SMCSO, 1.06 µM, 5.29 µM and 26.46 µM). Taking into consideration the levels of SMC and SMCSO in plant material, we choose three levels that could be physiologically relevant following the consumption of diets rich in cruciferous and allium vegetables. The precision was calculated from the relative standard deviation. The coefficient of variation (CV) (%) was less than 10% for intraday precision in both matrices. Interday precision was evaluated by analysing the same three levels concentration samples in urine and plasma by the same extraction and LC-MS/MS methods (n = 5 days). The CV (%) was <10% and <20% in urine and plasma, respectively. Precision and accuracy data are presented in Table 2.

#### 2.4.3. Carry-Over Effect

The carry over effect is a common encountered problem in the quantification of metabolites in biological samples by LC-MS/MS. In our study, we used Agilent 1290 series high performance auto sampler with an injection program to minimize carry-over effects. Responses for SMC and SMCSO analytes were approximately zero.

#### 2.4.4. Extraction Recovery and Matrix Effect

SMC (73.98 µM and 7.4 µM) and SMCSO (66.15 µM and 7.6 µM) were spiked in different matrices before carrying out our extraction method and LC-MS/MS analysis. We found a significant difference in the peak area of SMC and SMCSO in the different matrices using the electrospray ionization source. The extraction recovery was calculated as the ratio of the peak area of an analyte spiked prior to extraction to that from post-extraction samples. The extraction recovery in urine samples was 0.9 ± 0.1 and 0.8 ± 0.04 for SMC and SMCSO, respectively. Peak area ratios in plasma samples were 1.0 ± 0.1 for SMC and 0.7 ± 0.02 for SMCSO. The matrix effect was expressed as the peak area ratio of an analyte spiked post-extraction to that from 5% trichloroacetic acid (TCA) in water (SMC peak area ratio: 0.7 ± 0.05 in urine, 0.5 ± 0.1 in plasma; SMCSO peak area ratio: 0.6 ± 0.1 in urine, 0.5 ± 0.02 in plasma). Recovery values are presented in Table 2.

#### 2.4.5. Sample Stability

Stock solutions of SMC and SMCSO standard were prepared and kept at −20 °C for 7 days, and no changes were observed. The stability of the extracted samples was assessed following storage of the samples at 4 °C for 72 h. The CV (%) value was <10% for all the biological matrices tested in this study.

### 2.5. Quantification of SMC and SMCSO in Urine Samples

**Figure 2:**
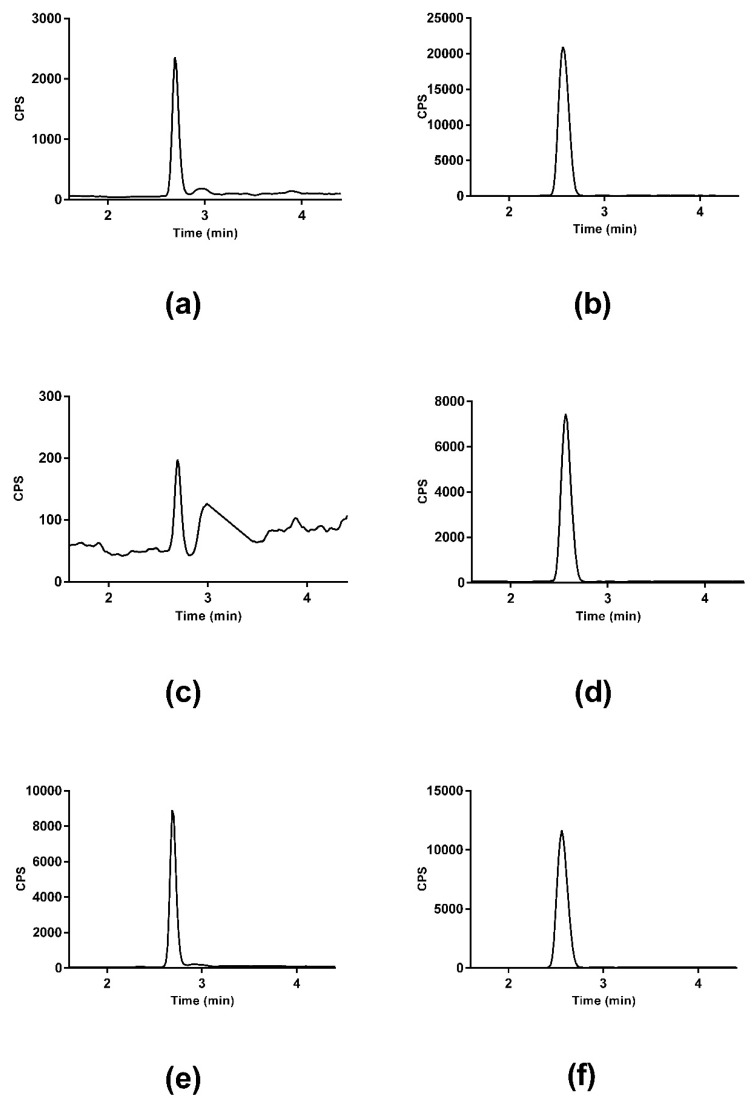
LC-MS/MS chromatograms of *S*-methyl-l-cysteine (SMC) and SMCSO in human urine. (**a**) SMC authentic standard in human urine; (**b**) SMCSO authentic standard spiked in human urine; (**c**) SMC detected in human urine; (**d**) SMCSO detected in human urine; (**e**) ^34^*S*-d_3_SMC internal standard in urine; (**f**) ^34^*S*-d_3_SMCSO internal standard in urine.

Serial dilutions for the calibration curve, two standards (SMC and SMCSO) were spiked in baseline urine samples to match the matrix of the urine sample. Figure 2a,b show SMC and SMCSO spiked in urine sample. Samples (n = 9) were de-proteinised with the addition of TCA and subsequently analysed on the LC-MS/MS. SMC and SMCSO peak were identified in all samples with retention times of 2.7 ± 0.01 and 2.6 ± 0.01 min, respectively. Figure 2c,d show the SMC and SMCSO peaks detected in a human urine sample. Figure 2e,f show the labelled internal standards of SMC and SMCSO in human urine. The calibration curve constructed from the standards with concentrations of 0–661.5 µM were linear with a correlation r^2^ value of >0.9995 for SMCSO and 0–739.75 µM were linear with a correlation r^2^ value of >0.9998. The average (n = 9) urinary concentration of SMCSO was 38.03 ± 21.28 μM, and SMC was 2.73 ± 0.61 μM. SMC levels were comparable with previous published reports [34].

### 2.6. Quantification of SMC and SMCSO in Plasma Samples

**Figure 3:**
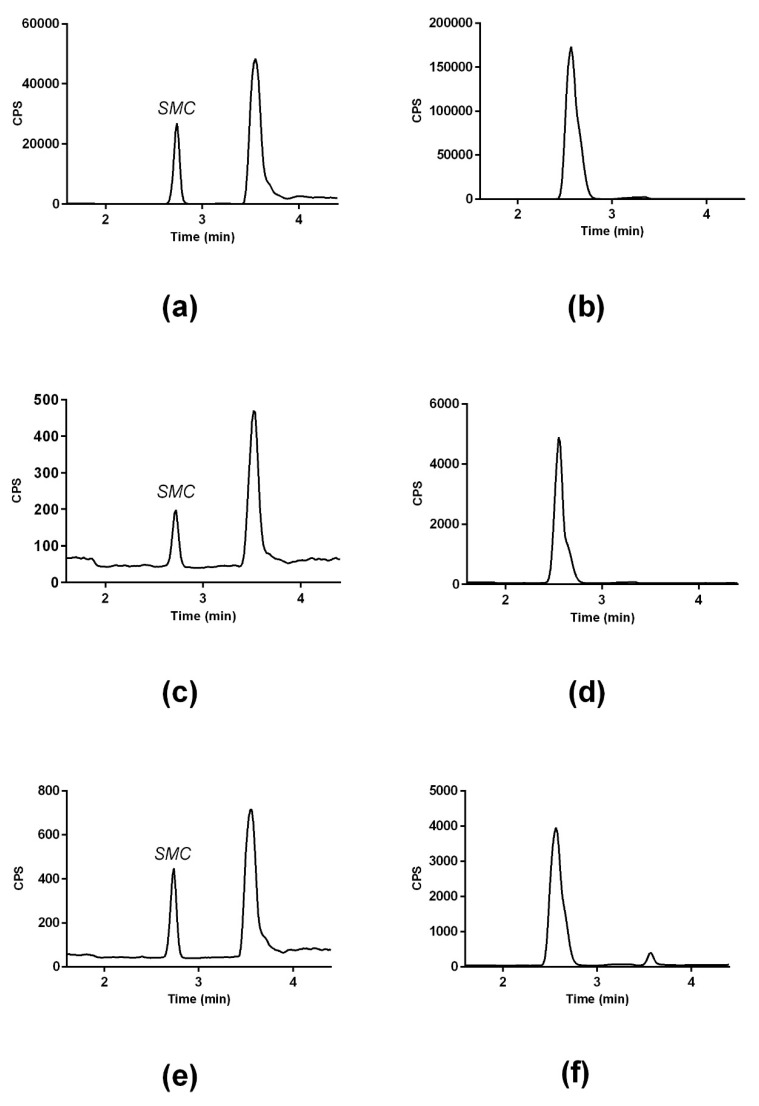
LC-MS/MS chromatograms of SMC and SMCSO in human plasma. (**a**) SMC authentic standard spiked in human plasma; (**b**) SMCSO authentic standard spiked in human plasma; (**c**) SMC detected in human plasma; (**d**) SMCSO detected in human plasma; (**e**) ^34^*S*-d_3_SMC internal standard in plasma; (**f**) ^34^*S*-d_3_SMCSO internal standard in plasma.

Commercially available human plasma samples (Sera lab) were used to spike to make a serial dilution for the calibration curve to match the matrix of the plasma sample. Figure 3a,b show SMC and SMCSO spiked in plasma sample. A 50% TCA solution was added to the samples to remove protein from the plasma samples and then analysed on the LC-MS/MS. SMC and SMCSO peak was detected in all samples with a retention time of 2.7 ± 0.01 and 2.6 ± 0.01 min, respectively. Figure 3c,d show the SMC and SMCSO peak detected in a human plasma sample. Figure 3e,f show the labelled internal standards of SMC and SMCSO in human plasma. The calibration curve for the standards ranged from 0 to 66.15 µM for SMCSO, and from 0 to 73.98 µM for SMC. The curve was linear with an r^2^ value of >0.9987. The average concentration of SMCSO in plasma (n = 5) was 4.12 ± 1.3 μM. Average plasma levels of SMC was 5.26 ± 1.35 μM as previously reported by Armstrong [37].

## 3. Discussion

LC-MS/MS-based analytical methods offer key advantages for the simultaneous detection of human metabolites in different biological matrices [38]. SMCSO was previously analysed in different plant materials by HPLC using derivatized methods which are less sensitive due to UV detection and require laborious sample preparation and long run time (~65 min) [31]. However, studies requiring the analysis of many samples would need to implement fast and simple sample preparation and non-derivatisation steps [11,21,22]. Methods without a derivatisation step have been developed to analyse SMCSO in plant material using HPLC-MS [26], HPLC [17,29], and LC-MS [27]. We aimed to develop a reliable, sensitive and accurate analytical method allowing researchers to run large batches of samples in a time-effective manner. We excluded the use of HILIC chromatography due to the length of time required for column equilibrium that would significantly extend run times. Therefore, we have developed a newly validated method which builds on the method of Kim [27] to analyse SMC and SMCSO in human specimens, using a different column and mobile phase composition. To our acknowledge, this is the first report describing a LC-MS/MS method for SMC and SMCSO analysis in human biological samples. The LC-MS/MS method described in this study allows analysis of very low level of SMCSO in plasma and urine samples with relatively easy sample preparation. Other authors have identified SMCSO in human samples, but they applied different analytical techniques such as NMR or HPLC [25,39]. Our analytical method for SMC and SMCSO analysis in urine and plasma was validated based on linearity, limit of detection, limit of quantification and accuracy in accordance with the Food and Drug Administration (FDA) guideline [35]. LOD, LOQ and accuracy values for SMC and SMCSO in urine and plasma are shown in Table 2. Correlation coefficients r^2^ of SMC and SMCSO in urine and plasma were >0.9987. The recovery values were satisfactory. To check matrix effects, SMC and SMCSO were spiked in different matrices after carrying out our extraction method as described in the Methods section. We found a significant difference in the peak area of SMC and SMCSO in the different matrices using the electrospray ionization source. To further investigate the matrix effect, a derivatisation method using dansyl chloride-derivatisation of urine and plasma samples was performed using Diode-Array Detection (DAD) [31]. We observed similar peak areas when SMCSO was derivatised confirming matrix effect or ion suppression due to ionization effect for interference compounds (data not shown). Therefore, the matrix match calibration is crucial for quantifying SMC and SMCSO in different biological matrices by using this LC-M/MS method.

Another important aspect to take into consideration is the use of a suitable internal standard. DL-Norleucine was previously used as internal standard to quantify SMCSO in plant extracts [29]. We also used this compound in our method; however, an interfering peak was appearing at the same retention time and with the same fragmentation pattern. For this reason, dl-Norleucine was not used for further analyses. An alternative internal standard is *S*-butylcysteine sulfoxide (BCSO) used by Kubec and colleagues for its stability, solubility and chemical properties [20]; however, BCSO is not commercially available. Therefore, ^34^Sd_3_-labeled two isotope labelled internal standards were synthesised in our laboratory to use to analyses SMC and SMCSO in biological human samples for LC-MS/MS analysis.

Finally, data obtained from the analysis of SMCSO in plasma samples by following the extraction method described in this study were compared to those reported by Barry and colleagues in plasma collected from kale-fed lambs [40]. The use of 50% TCA solution enhance the mass spectroscopy signal which resulted in high sensitivity allowing the detection of low level of SMCSO in plasma.

## 4. Materials and Methods

### 4.1. Chemical and Reagents

SMCSO (>94%) was purchased from LKT laboratories (St. Paul, MN, USA). *S*-methyl-l-cysteine (>98%), trichloroacetic acid (TCA) (>99%) and heptaflurobutyric acid (>99.5%) were purchased from Sigma^®^ (Gillingham, UK). All chemical solvents with high purity grade were used for LC-MS analysis: Ammonium acetate (>99%) was purchased from Fluka. Purified water was obtained from the Milli-Q^®^ Integral Water Purification System (Millipore Advantage, Watford, UK).

### 4.2. Isotope Labelled Internal Standards (34S-Trideuteromethylcysteine and 34S-Trideuteromethylcysteine Sulfoxide)

^34^*S*-Trideuteromethylcysteine (^34^*S*-d_3_SMC) and ^34^*S*-trideuteromethylcysteine sulfoxide (^34^*S*-d_3_ SMCSO) were synthesised in house. ^34^*S*-Trideuteromethylcysteine was synthesised via treatment of methyl *N*-(tert-butoxycarbonyl)-*O*-tosyl-l-serinate with ^34^*S*-trideuteromethylthiomethoxide, followed by deprotection with trifluoroacetic acid (unpublished data). ^34^*S*-Trideuteromethylcysteine sulfoxide was synthesised by oxidation of ^34^*S*-trideuteromethylcysteine with hydrogen peroxide (unpublished data). SMCSO was obtained as a mixture of racemic diastereomers. These compounds were fully characterised by NMR and LC-MS. ^1^H-NMR spectra were recorded on a Bruker Avance NMR spectrometer (Bruker BioSpin GmbH, Rheinstetten, Germany) equipped with a cryoprobe (TCI) and operating at 600 MHz. Trimethylsilylpropanoic acid (TSP) was used as a reference. LC-MS analysis was performed on an Agilent 1100 system (Agilent Technologies, Santa Clara, CA, USA) equipped with an MSD SL single quadrupole mass spectrometer (Agilent Technologies, Santa Clara, CA, USA), using a Phenomenex Luna 100 mm × 4.6 mm, 3 µm column eluted with an ammonium acetate pH 5, acetonitrile gradient. These compounds were used as an internal standard for the analysis of SMC and SMCSO in human urine and plasma.

### 4.3. Human Body Fluids

For purposes of method development and with local ethical approval, plasma (n = 5) and urine (n = 10) samples were collected from healthy human subjects. For matrix-match calibration, pooled human plasma (male and female, 18–65 years) was obtained from Sera Laboratories Internationals (USA). Plasma samples (100 µL) were mixed with 50% trichloroacetic acid (20 µL) and 10 µL of 10 µg/mL concentration of mixed internal standards (^34^*S*-d_3_SMC and ^34^*S*-d_3_SMCSO). The mixture was vortexed for 30 s, and kept on ice for 5 min. After centrifugation at 17,000× *g* for 10 min (4 °C), supernatants were transferred to HPLC vials for analysis by LC-MS/MS. Supernatants were transferred to HPLC vials and analysed by the LC-MS/MS on the same day. After filtration by using a ministart Sterile-ED 0.20 µm filter, urine samples (10 µL) were added to 5% TCA (80 μL) and 10 µL of 10 µg/mL concentration of mixed internal standards. The mixture was vortexed for 30 s and kept on ice for 5 min. Samples were then centrifuged at 13,000× *g* for 10 min (4 °C). Supernatants were transferred to HPLC vials and analysed by the LC-MS/MS on the same day.

### 4.4. LC-MS/MS Analysis

SMC and SMCSO authentic standards were reconstituted in Milli-Q^®^ water to prepare stock solutions at the concentration of 7.4 and 6.6 mM, respectively. All stock solutions were kept at −20 °C. A standard curve was produced from stock solutions in the relevant matrix (urine, pooled plasma). A standard curve was produced with serial dilutions from the highest concentration (739.75 µM for urine; 73.98 µM for plasma) to 0 for SMC and (661.46 µM for urine; 66.15 µM for plasma) to 0 for SMCSO. All serial dilutions were prepared prior to each run. Agilent 6490 Triple Quad MS mass spectrometer equipped with an Agilent 1290 HPLC system (Agilent Technologies, Santa Clara, CA, USA) was used for the analysis of SMCSO and SMC. The LC flow rate was 0.1 mL/min. The column used for the analysis was an Agilent SB-AQ 1.8 µM (100 mm × 2.1 mm) C18 column with an Agilent Zorbax guard column. The column temperature and auto sampler were maintained at 20 °C and 4 °C, respectively. 2 µL was used for the injection volume. Extracted samples were analysed using 10 mM ammonium acetate + 0.05% heptaflurobutyric acid (HFBA) in water (mobile phase A) and 10 mM ammonium acetate + 0.05% HFBA in 90% methanol (mobile phase B). The gradient was started with 0% B, increased 3% B within 4 min, after washing for 2 min and equilibration was for another 2 min. The total run was 8 min. The 6490 MS/MS system was equipped with an electrospray ionization (ESI) source operated in positive-ion detection mode. Nitrogen gas was used for nebulation, desolvation, and collision. The analytes were monitored in multiple-reaction monitoring (MRM) mode. The MRM precursor, product ions and collision energy were optimized by Agilent optimizer software. The transitions of precursor ions to product ions (*m*/*z*) and some optimized MS operating parameters of the analyte are described in Table 1. The source parameters were: Gas temperature of 200 °C with a gas flow of 16 L/min, a sheath gas temperature of 300 °C with a sheath gas flow of 11 L/min, a nebuliser pressure of 50 psi and capillary voltage of 3500 V for positive polarity, Nozzle Voltage 1000 V. The iFunnel parameters were: High pressure radio frequency (RF) of 150 V and low-pressure RF of 60 V. The LC eluent flow was sprayed into the mass spectrometer interface without splitting. Identification was achieved based on retention time of authentic SMC and SMCSO standards and by product ions monitor.

### 4.5. Analytical Validation

#### 4.5.1. Linearity

Authentic standards were spiked in different matrices (urine and plasma) to construct calibration curves for SMC and SMCSO analysis. The concentrations versus peak area ratio (analyte peak area /internal standard peak area) were plotted to obtain the calibration curves.

#### 4.5.2. Sensitivity

Diluted solutions of SMC and SMCSO in each matrix was injected to get LOD and LOQ values. LOD was calculated as signal to noise ratio at least three times higher than the baseline noise. LOQ was calculated at a signal to-noise ratio 10 times higher than the baseline noise of this compound.

#### 4.5.3. Precision and Accuracy

Intraday precision and were accuracy calculated by analysis of replicate spiked urine and plasma samples at concentrations of 1.18 (L), 29.59 (M) and 739.75 (H) µM in human urine and 1.18 (L), 5.92 (M) and 29.59 (H) µM in human plasma for SMC, 1.06 (L), 26.46 (M) and 661.46 (H) µM in human urine and 1.06 (L), 5.29 (M) and 26.46 (H) µM in human plasma for SMCSO (n = 5 at each level) on the same day. To assess the inter day precision and accuracy, replicate spiked same levels of samples (n = 2) were analysed on five different days. The precision was calculated from the relative standard deviation (R.S.D. %) of the replicate analyses. A R.S.D. % of 20% in biological sample was deemed acceptable for precision. Accuracy was calculated by comparison of expected concentrations with the measured concentrations of the spiked samples.

#### 4.5.4. Carry-Over Effect

Acidified water was injected to assess carry-over effects after an injection of the highest concentration of the SMC and SMCSO standards. Agilent 1200 series high performance auto sampler with an injection program was used to minimize carry-over effects.

#### 4.5.5. Extraction Recovery and Matrix Effect (or Ion Suppression)

The post-extraction spike method as indicated by RSC guideline for LC-MS measurements was used to assess the matrix effect [35]. Two concentrations of SMC (73.98 µM and 7.4 µM) and SMCSO (66.15 µM and 7.6 µM) were spiked in different matrices in extracted samples: (1) 5% TCA in water and blank urine was used to assess the LC-MS/MS matrix effect for urine analysis, (2) 5% TCA in water and blank plasma was used to assess the matrix effect for plasma analysis. The same procedure was followed for recovery assessment except for SMC and SMCSO which was spiked before extraction of samples.

### 4.6. Data Analysis

Data files were exported and analysed on Agilent MassHunter Quantitative analysis B.06.00/Build 6.0.388.0 (Agilent Technologies). The software integrates the peak area for the metabolites which is then exported as an Excel document. The concentration of the metabolites was calculated using the equation of the standard curve and the peak area ratio (analyte peak area /internal standard peak area) of the metabolites.

### 4.7. Ethical Considerations

All procedures performed involving human participants were in accordance with the ethical standards of the institutional and/or national research committee and with the 1964 Helsinki declaration and its later amendments or comparable ethical standards. The collection, storage and use of all human samples was carried out within the terms of the Human Tissue Act 2004 (Human Tissue Authority).

## 5. Conclusions

In conclusion, the current method uses stably labelled internal standards to reduce the matrix effect allowing accurate measurement of SMC and SMCSO in human biological matrices. We are currently working on the application of this method for the analysis of SMC and SMCSO in cell culture and tissue samples of human origin. It is envisaged that this method could be used for bioavailability studies for the quantification of SMC and SMCSO delivered in our body by SMC/SMCSO enriched diets, novel foods and supplements. Furthermore, the application of this analytical tool to ex vivo and in vitro models will help carrying out mechanistic studies to elucidate SMC and SMCSO biological profile.

## Figures and Tables

**Table 1:** Liquid chromatography-mass spectrometry (LC-MS)/MS parameters of *S*-methyl-l-cysteine sulfoxide (SMCSO).

Analyte	Retention Time (mins)	Precursor Ion (*m*/*z*)	Product Ion (*m*/*z*)	Collision Energy	Cell Accelerator Energy	Polarity
SMCSO	2.6	152.19	87.9	4	4	Positive
SMCSO	2.6	152.19	69.9	16	4	Positive
SMCSO	2.6	152.19	42.1	20	4	Positive
SMC	2.7	136.19	119.1	10	4	Positive
SMC	2.7	136.19	47	34	4	Positive
^34^*S*-d_3_SMCSO	2.6	157.05	87.9	8	4	Positive
^34^*S*-d_3_SMCSO	2.6	157.05	42.1	28	4	Positive
^34^*S*-d_3_SMC	2.7	141.06	124	10	4	Positive
^34^*S*-d_3_SMC	2.7	141.06	42.1	30	4	Positive

**Table 2:** Validation data in human plasma and urine.

Analyte	R^2^	Precision (n = 5) (Intra-Day) R.S.D. %			Precision (n = 5) (Inter-Day) R.S.D. %			Accuracy (% Recovery)			LOD (µM)	LOQ (µM)
		L	M	H	L	M	H	L	M	H		
SMC (Urine)	0.9998	9.44	2.00	5.13	9.19	8.17	5.28	82.8	101	101	0.08	0.24
SMC (Plasma)	0.9987	8.8	6.8	7.2	11.29	7.81	7.16	97.7	97.0	95.49	0.04	0.12
SMCSO (Urine)	0.9995	5.49	5.14	5.43	14.36	7.11	5.50	100.4	105.98	101.2	0.03	0.09
SMCSO (Plasma)	0.9989	8.86	2.1	4.8	19.80	10.69	6.72	99.64	99.38	97.77	0.02	0.06
